# Burden of Pulmonary Rifampicin-Resistant Tuberculosis in Kajiado, Kenya: An Observational Study

**DOI:** 10.3390/microorganisms11051280

**Published:** 2023-05-13

**Authors:** Paolo Cattaneo, Caleb Mike Mulongo, Gianfranco Morino, Maria Vittoria De Vita, Gabriele Paone, Simone Scarlata, Salome Kinyita, Hillary Odhiambo, Cristina Mazzi, Federico Gobbi, Dora Buonfrate

**Affiliations:** 1 Department of Infectious, Tropical Diseases and Microbiology, IRCCS Sacro Cuore Don Calabria Hospital, Negrar di Valpolicella, 37024 Verona, Italy; cristina.mazzi@sacrocuore.it (C.M.); federico.gobbi@sacrocuore.it (F.G.); dora.buonfrate@sacrocuore.it (D.B.); 2 Independent Researcher, Nairobi 79644-00200, Kenya; mike_mulongo@yahoo.com; 3 World Friends Amici del Mondo Onlus, Ruaraka Uhai Neema Hospital, off Thika Highway, Nairobi P.O. Box 39433-00623, Kenya; gianfranco.morino@worldfriendskenya.org (G.M.); mariavittoria.devita@worldfriendskenya.org (M.V.D.V.); gabriele.paone@worldfriendskenya.org (G.P.); 4 Unit of Internal Medicine, Respiratory Pathophysiology and Thoracic Endoscopy, Fondazione Policlinico Universitario Campus Bio Medico, 00128 Rome, Italy; s.scarlata@policlinicocampus.it; 5 Independent Researcher, Gatundu 324-01030, Kenya; kinyita@gmail.com; 6 Independent Researcher, Nairobi 00500, Kenya; hilljairus@gmail.com

**Keywords:** tuberculosis, rifampicin resistance, active-case finding, Kenya, Kajiado, HIV coinfection

## Abstract

Background: Rifampicin resistance (RR) is a major challenge in the clinical management of tuberculosis (TB), but data on its prevalence are still sparse in many countries. Our study aimed at estimating the prevalence of RR-TB in Kajiado County, Kenya. Secondary objectives were to estimate the incidence of pulmonary TB in adults and the rate of HIV-TB coinfection. Methods: We conducted an observational study in the context of the ATI-TB Project, carried out in Kajiado. The project was based on an active-case-finding campaign implemented with the aid of village chiefs, traditional healers and community health volunteers. Diagnosis relied on Xpert MTB/RIF, including a mobile machine that could be used to cover areas where testing would otherwise be difficult. Results: In sum, 3840 adults were screened for active TB during the campaign. RR cases among all TB diagnoses were 4.6%. The annual incidence of pulmonary TB among adults was 521 cases per 100,000 population. The rate of HIV coinfection was 22.2% among pulmonary TB diagnoses. Conclusion: The prevalence of RR-TB was four times that what could be inferred from official notifications in Kajiado, and higher than overall prevalence in Kenya. In addition, our estimate of incidence of pulmonary TB in adults in Kajiado significantly differed from cases notified in the same area. In contrast, the rate of HIV coinfection was in line with national and regional data. TB diagnostic capability must be strengthened in Kajiado to improve patients’ management and public health interventions.

## 1. Introduction

Tuberculosis (TB) is one of the leading causes of death at a global level, ranking first for mortality rate from a single infectious disease from 2015 until the emergence of the COVID-19 pandemic [1,2,3,4,5,6,7].

About 10.6 million cases of active TB disease occurred in 2021 [1], with about 1.6 million deaths. The biggest burden of the disease hits low- and middle-income countries (LMICs), which register about 95% of TB deaths globally [2,8,9]. Compared to a case–fatality rate that is no more than 5% in high-income countries, many African countries present a lethality rate well above 20% [1]. To further complicate this picture, the burden of TB in LMICs is also affected by a high prevalence of HIV coinfection, which makes the clinical management more challenging [1].

Multidrug-resistant (MDR) and rifampicin-resistant (RR) TB have an even higher impact on TB management than HIV coinfection, representing one of the major threats to the global TB control efforts. Estimates of the global prevalence and incidence of MDR and RR-TB have been hampered by the scarce availability of drug-susceptibility testing, due to limited access to diagnostic tests in most endemic countries [10,11]. Despite the wider availability of diagnostic tests during the last 5 years [1,2,3,4,12], a significant gap between the reported and the estimated RR-TB cases is still present [1].

The WHO Consolidated Guidelines on TB Diagnosis advocate for a wide implementation of molecular biology and for the use of Xpert MTB/RIF as first-line diagnostic test for TB, rather than smear microscopy/culture [13]. Among the advantages of Xpert MTB/RIF are higher sensitivity than microscopy for pulmonary TB, results within 2 h, and information about rifampicin resistance [13]. A wide deployment of Xpert MTB/RIF is supposed to narrow the gap between the true incidence of TB and the number of notified cases, which is of pivotal importance [1,14,15,16,17].

Kenya is one of the 30 high TB- and high HIV/TB-burden countries listed by the WHO [18]. The epidemiology of TB in Kenya has been well investigated at country level, and also through a recent national TB prevalence survey [19]. According to the latest WHO estimates, TB incidence was 251 per 100,000 population in 2021, with an HIV coinfection rate of 24%. Of the total notified cases, 87% were pulmonary TB (PTB) cases; of these, only 63% were bacteriologically confirmed [1]. With regard to MDR/RR-TB cases, only 63% of bacteriologically confirmed new PTB cases and 73% of previously treated PTB cases were tested for rifampicin resistance in 2021. The proportion of RR-TB among them was 1.0–1.1%, equivalent to 0.6–0.7% of all notified PTB cases [1].

TB epidemiological data are much scanter at regional level, and thus possible significant differences might exist across the country. Often, notifications are the only available data to conduct subnational estimates [19,20].

This applies to Kajiado County, one of the vastest counties in Kenya, bordering Tanzania. The county, which is divided into the five sub-counties of North, West, Central, East and South Kajiado, includes a small but dense urban area (limited to Kajiado North), and a rural area (other four sub-counties) [21].

At the beginning of 2021, there were only three health centers equipped with Xpert MTB/RIF systems in Kajiado for a population of a little more than 1 million inhabitants [22]. Though official notifications [23] can be used to estimate the epidemiological situation of TB in Kajiado (Table S1), these data presumably represent an underestimation of the true incidence of the disease. Other studies have already demonstrated that TB is largely underdiagnosed in rural communities in Kenya [24].

The aim of this study was to estimate the proportion of RR-PTB cases in Kajiado among all new and relapsed PTB cases. Secondary objectives were:

(i) to measure the rate of HIV coinfection;
(ii) to estimate the incidence of PTB among adults (people aged ≥ 15 years) in Kajiado.

## 2. Materials and Methods

### 2.1. Study Design

This was an observational study performed in the context of the master project Awareness, Tradition and Innovation to Fight TB (ATI-TB), carried out in Kajiado County by the Italian NGO World Friends Onlus. Briefly, the ATI-TB project started in June 2021 and consisted of a “raising awareness campaign” in Kajiado’s urban and rural area, immediately followed by active-case-finding (ACF) activities. The project actively involved community health volunteers, village chiefs and traditional healers in the ACF activities. Three new Xpert MTB/RIF systems were supplied in Kajiado by the ATI-TB project.

All Kajiado’s sub-counties except Kajiado South were included in the project, and most activities were carried out in Kajiado North and Kajiado West. A map of all the sites involved in the project is shown in Figure S1.

Data included in this sub-study were collected for people tested from 1 June 2021 to 31 May 2022.

### 2.2. Study Population

The study population consisted of all the participants in the ATI-TB project that were tested for PTB (from here on, “presumptive PTB cases”) by Xpert MTB/RIF assay (Cepheid). Participants included both people tested during the awareness and ACF campaigns (i.e., active-case finding) and the so-called walk-in patients. The latter were people who spontaneously accessed a local health facility because of symptoms (passive-case finding) and got their sputum tested using one of the two GeneXpert machines purchased by the ATI-TB project and installed in Ngong (Kajiado North) and Oltepesi (Kajiado West). These machines were used to test both the locally collected sputum samples and those sent from other health centers of the area. The third GeneXpert machine bought for the project was used as a mobile Xpert MTB/RIF for the ACF activities.

### 2.3. Testing Strategy and Sample Collection

People who participated in ACF activities underwent clinical examination by an ATI-TB clinical officer or nurse. Any participant reporting one or more of the symptoms listed in the questionnaire recommended by the last Kenyan TB guidelines [25] (cough of any duration, hotness of body or fever >37.5 °C, drenching night sweats, unintended weight loss or BMI less than 18.5, chest pain) was considered a presumptive PTB case, and was invited to supply a sputum sample. People presenting with hemoptysis were also tested. The sputum was tested by Xpert MTB/RIF either immediately on-site or—when it was not possible to bring the GeneXpert machine on-site due to logistical reasons—once back in Ngong, within 24 h from collection. The sputum was collected in a sterile Falcon tube, and if needed, stored at 4 °C before being tested by Xpert MTB/RIF. A PTB case was defined as a person with a positive Xpert MTB/RIF sputum test. If the result of the test was invalid or indeterminate, a new sputum sample was collected for retesting, as an attempt to allow proper classification.

Regarding the walk-in patients, the screening strategy was also based on the aforementioned national TB guidelines [25]. However, as walk-in patients were not evaluated by ATI-TB staff, but by different clinicians working in the many health centers that referred to the Xpert MTB/RIFs of the project for TB testing, a homogeneous fulfillment of these indications cannot be proved.

The awareness and ACF campaigns were conducted repeatedly at the same site once a month or every two months. For some communities in Kajiado Central and East, the ACF campaigns started some months later than June 2021.

All TB-positive cases were tested for HIV infection by rapid test, in line with the national guidelines [25].

### 2.4. Data Collection

Clinical, epidemiological and microbiological data were collected for all participants from the mandatory lab-request forms filled in at the moment of sputum collection, as well as from test registers and medical records. Age, sex, sub-county of origin, type of patient (whether identified by ACF or walk-in patient), date of sputum collection, and Xpert MTB/RIF result were collected from all the presumptive cases. In addition, HIV status and resistance status to rifampicin were collected from the PTB cases.

Furthermore, the cumulative number of people who participated in the awareness and ACF campaigns is available, divided by urban and rural areas; age and sex proportion of these subjects is also cumulatively available.

### 2.5. Data Analysis

All collected data were anonymized and entered in an Excel database with access restricted to the study PI and to ATI-TB staff.

The primary outcome was calculated as the number of RR-PTB cases over all PTB cases tested for rifampicin resistance. Data from both ACF-detected PTB cases and walk-in PTB cases were used to measure the rate of HIV confection, calculated as the number of HIV+ cases over all PTB cases.

The incidence of PTB in Kajiado was calculated considering positive PTB cases in people aged over 14 years and detected by ACF; the denominator was the total number of adults (≥15 years old) who participated in the awareness and ACF campaigns. TB incidence was also estimated by urban and rural areas.

All data were analyzed with R Core Team 2022 [26] (Version 4.2.1). Categorical data are presented as absolute numbers and proportions, while continuous variables are presented as medians and interquartile ranges. The χ2 test with simulated *p*-value was used to compare proportions between groups of interest, whereas the exact binomial test or the χ2 test of homogeneity was used to compare the proportions observed between our project and Kenya’s and Kajiado’s estimates. The Firth multivariable regression model was used to test associations between RR-TB or TB (adult cases, ≥15 years) and variables of interest (age classes, sex and sub-county of origin). Results were considered statistically significant if the two-tailed *p*-value were less than 0.05. All estimates are presented with 95% confidence intervals.

## 3. Results

### 3.1. Characteristics of the Population

**Figure 1:**
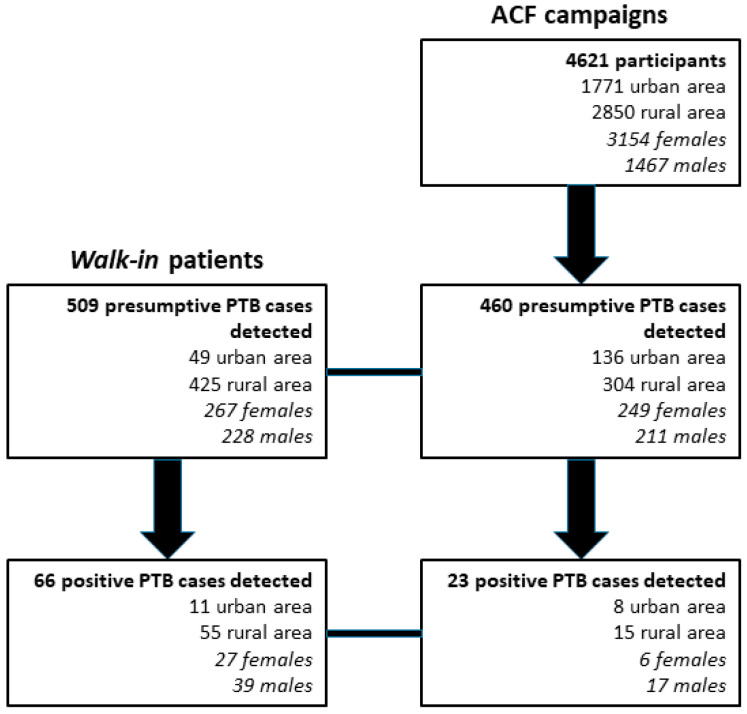
Study flow.

People that participated to the awareness and ACF campaigns numbered 4621, while walk-in participants numbered 509. The study flow is shown in Figure 1.

Although individual data about sex and area of origin of some presumptive cases were missing, information about them was cumulatively available for all participants.

The age distribution and the male:female ratio among the adult participants (≥15 years) to the ACF campaigns are depicted in Table 1 and compared to Kajiado’s adult population (further data about Kajiado’s general population are shown in Table S1). Participants aged ≥15 years numbered 3840, with 1583 of them coming from an urban area and 2257 from a rural area. The proportion of women was 71%.

Thus, among the adult participants to the awareness and ACF campaigns of the ATI-TB project, we observed a significant underrepresentation of participants from the youngest age-group (15–34 years) and an underrepresentation of males compared to Kajiado’s general adult population (both *p* < 0.01).

For both ACF-detected and walk-in patients, 969 participants with presumptive PTB were tested by Xpert MTB/RIF, of whom 89 were diagnosed with PTB.

The demographic characteristics of all the presumptive and positive PTB cases are depicted in Table 2.

Of note, women represented around 54% of presumptive cases, but only 37% of PTB cases.

Regarding the sub-county of origin, 80% of the presumptive PTB cases and 79% of the PTB cases were from rural areas. Among presumptive cases, the proportion of people coming from rural areas was 69% in the ACF-detected patients vs. 90% in walk-in patients (p < 0.01), while it was 65% in ACF-patients vs. 83% in walk-in patients among the positive PTB cases (p = 0.13). People coming from Kajiado North and Kajiado West were more represented, because two of the three Xpert MTB/RIFs purchased by the ATI-TB project were installed in these sub-counties, while the remaining one was used for ACF activities. Hence, walk-in patients were more likely to come from these areas than from Kajiado Central and Kajiado East. However, focusing on ACF subjects, the proportion of people coming from East and Central Kajiado was higher (17% and 5%, respectively), even if still lower than those from North and West Kajiado.

### 3.2. Study Outcomes

RR-TB was tested in 87 out of 89 PTB cases, as for two participants *Mycobacterium tuberculosis* complex DNA was found only in traces in the sputum, not allowing reliable Xpert MTB/RIF evaluation of rifampicin resistance. For both participants, the sputum collection was done twice with the same result.

Four cases of RR-TB were found, corresponding to a prevalence of 4.6% (95% CI: 1.3–11.4%). None of the four individuals belonged to the same family cluster or had known contact with each other.

On multivariable analysis, we did not find any association between the presence of RR-TB and age, sex, or area of origin.

HIV status was available for 63 of 89 PTB cases, and the HIV-coinfection rate was 22.2% (95% CI: 13.1–34.8%).

Twenty PTB cases among 3840 adult participants (≥15 years) in the awareness and ACF campaigns were diagnosed; hence, the estimated incidence of PTB cases was 521 (95% CI: 327–819) per 100,000 adult population. Finally, annual PTB incidence was 442 (95% CI: 214–910) and 576 (95% CI: 337–983) per 100,000 adult population in urban and rural areas, respectively.

On multivariable analysis, male sex was significantly associated with a diagnosis of PTB (*p* < 0.01).

## 4. Discussion

The present study demonstrated a high proportion (4.6%) of RR-PTB in Kajiado. Both prevalence of RR and incidence of PTB were higher than official estimates. Conversely, HIV coinfection was in line with national and regional data.

The prevalence of RR-PTB was more than four times that of Kajiado’s pre-COVID figures (1.2% among the bacteriologically confirmed and notified PTB cases in 2019, *p* = 0.02), as well as significantly higher than Kajiado’s prevalence, according to official data, in the same study period (1.0% among bacteriologically confirmed-notified PTB cases in 2021–2022, *p* = 0.02) [23]. Moreover, RR-TB prevalence found in our study was significantly higher than prevalence in the whole country, according to official data of 2021 among bacteriologically confirmed PTB patients (1.1%, *p* ≥ 0.02) [1].

There are some explanations for these discrepant results. First, the socioeconomic and geographical context: Kajiado is a poor region within Kenya, mainly rural, with a lot of hard-to-reach areas, where the available TB services face many logistical issues. Secondly, the low number of Xpert MTB/RIFs in the county, coupled with a very low/inexistent capability of performing mycobacterial culture, hampers diagnosis of RR/MDR-TB. Finally, the disruption of TB services due to the COVID-19 pandemic [1] might have caused an increase in the prevalence of rifampicin resistance, not yet detected by official notifications.

The WHO removed Kenya from the global list of countries with high MDR/RR-TB burden in 2021 [18,27]. This was based on data from national estimates of Kenyan MDR/RR-TB prevalence and incidence, which showed an improvement in the epidemiological situation at national level. However, it is worthy of note that what is true at national level might not mirror the local epidemiology within a county, as the results of this study suggest. Consequently, resources to tackle MDR/RR-TB might be reduced at Kenyan level as a consequence of this countrywide success, despite the same not being achieved in some regions. This might lead to further worsening of the local epidemiology. Thus, the results of this study highlight the need to monitor the level of rifampicin resistance in Kajiado and to boost financial resources to address this issue in the county.

Easy access to Xpert MTB/RIF was of paramount importance to obtain reliable data in our study. Despite sputum testing by this tool being advocated by WHO guidelines and by the national TB guidelines [13,25], only three Xpert MTB/RIFs were present in the whole county prior to the implementation of the ATI-TB project, which means only about one Xpert MTB/RIF per 350,000 population. Furthermore, a third of all TB diagnoses in the county are still based on clinical evaluation, again in great contrast to indications of Kenyan guidelines [23,25]. The widespread use of highly sensitive diagnostic tools, such as Xpert MTB/RIF, is recommended to detect subclinical cases [28]. This has been demonstrated in a recent study that showed that detecting subclinical cases would be an asset for preventing spread of TB in the community, as long as a relevant proportion of cases that would benefit from treatment were reached and would not be diagnosed based on clinical evaluation [29]. Our results confirm a clear gap between the real incidence of PTB and the one inferred by notifications. Indeed, the incidence we found (521 PTB cases per 100,000 adult population) is significantly higher than what could be inferred through the official notifications in Kajiado, both in the pre- and post-COVID period (all *p* < 0.001). From TIBU (National TB Reporting Platform) data of 2019, the incidence of PTB among adults in Kajiado (clinical diagnoses included) appeared to be 201 per 100,000, while in 2021–2022 it was 172 per 100,000. Considering bacteriologically confirmed PTB diagnoses among adults, Kajiado’s incidence in these two periods was even lower—146 and 126 per 100,000 respectively [23]. Probably both underdiagnosis and underreporting account for this gap, meaning that diagnostic capability, as well as regularity and accuracy of notifications, should be strengthened.

Notifications in Kajiado showed a clear decrease after the onset of the COVID-19 pandemic [23]. Therefore, it cannot be excluded that part of the gap with official notifications might be explained by both a drop in notifications during the pandemic and a real increase in the incidence of TB owing to the disruption of TB services/surveillance.

The incidence we found is not significantly different from WHO estimates of incidence in the adult population in Kenya for 2021: 368 per 100,000 adult population (*p* = 0.15) [1,30]. However, these figures represent all TB diagnoses in Kenya, including extrapulmonary cases. According to notified cases, the proportion of pulmonary TB is 87% [1]. Based on this, PTB incidence would be 320 per 100,000 adult population at a national level, which is instead statistically different (lower) than the PTB incidence found in our study (*p* = 0.04). Further studies are needed to confirm this result, given its important public health implications at local level.

HIV/TB-coinfection rate was 22.2% among PTB cases in our study, virtually identical to what emerged from Kajiado’s notifications both pre-COVID (23.8% in 2019, *p* = 0.88) and post-COVID (23.4% in 2021–2022, *p* = 0.94), as well as from Kenyan estimates on TB diagnoses (24% in 2021, *p* = 0.85) [1,23]. Hence, this result confirms the well-established Kenyan data and does not put Kajiado in a different position from the rest of the country.

The study has some strengths and limitations.

The ain strength was the deployment of one the most sensitive rapid diagnostic tools to test sputum (Xpert MTB/RIF), together with active-case finding and well-developed awareness campaigns, constantly repeated over time and inclusive of the local authorities. This should permit minimization of underdiagnosis and make overlooked TB cases emerge. Although other ACF programs for TB have been carried out in the region, this is, to the best of our knowledge, the first to use its results to infer general data about the local epidemiology. Only the national TB-prevalence survey conducted in Kenya in 2015 gathered data about Kajiado, but it was not powered to allow estimates about the regional epidemiology [20].

Some limitations need to be underpinned. Firstly, although the proportion of RR-TB was statistically higher than Kajiado’s and Kenya’s, we found very few cases of RR-TB, translating into a wide confidence interval.

Secondly, we decided to consider only adults (≥15 years) to estimate the incidence of PTB, since the proportion of children (0–14 years) among the participants in the awareness and ACF campaigns was very low compared to the weight of the same age-group among the general population in Kajiado [21]. Moreover, the risk of active TB in children is known to be substantially lower than in older ages [1,19,31,32]. Taken together, the inclusion of children in the calculation of incidence would have biased the results. Of note, children were included to estimate the primary outcome. In our cohort, people aged 15–34 years and male subjects were underrepresented compared to Kajiado’s general population, and this difference is likely to result in underestimation of PTB incidence in our sample, because young male adults are the major group at risk of PTB in Kenya and in general for LMICs [1,19]. Another limitation is that chest X-ray was not included in the project as a screening tool; therefore, we could not identify active PTB cases that were completely asymptomatic. The use of TB LAM (lipoarabinomannan antigen test in urine) was not considered, despite being recommended by the national guidelines for HIV people [25]; thus, some HIV-coinfected patients might have been overlooked. Finally, the ACF campaigns in Kajiado East and Kajiado Central started a little later than June 2021; nonetheless, the fact that only a minority of participants were from these sub-counties makes it unlikely to have highly affected our results.

It must be considered that people in very good health may tend to avoid participating to awareness campaigns, not feeling touched by the issue of TB or fearing stigma; however, this limitation is common to any TB prevalence survey, and in our study, the involvement of traditional healers and community caregivers was also meant to limit this issue as much as possible.

Finally, the ATI-TB project was not set in Kajiado South; therefore, our results might not be generalizable to this sub-county.

## 5. Conclusions

This study found a prevalence of RR-TB among PTB cases in Kajiado higher than both national estimates and regional data derived from official notifications. The incidence of PTB among adults in Kajiado was also higher than what can be inferred by the official TB notifications in the county. HIV-coinfection rate is similar to the data already available at national and regional level. Wider availability of Xpert MTB/RIF testing and active surveillance would be needed in peripheral regions to obtain reliable data on RR-TB prevalence and monitor its spread within the country.

## Figures and Tables

**Table 1:** Age and sex proportions among adults in ACF campaigns and in Kajiado.

	ACF Campaign Participants		Kajiado’s General Population	
	Age Percentage	Male: Female Ratio	Age Percentage	Male: Female Ratio
15–24 years	20.3%	0.37:1	35.8%	0.85:1
25–34 years	27.1%	0.37:1	30.6%	1.04:1
35–44 years	21.3%	0.42:1	17.0%	1.18:1
>44 years	31.3%	0.43:1	16.6%	1.09:1

**Table 2:** Characteristics of the study participants.

	Presumptive PTB Cases			PTB Cases		
	All (n = 969)	ACF (n = 460)	Walk-Ins (n = 509)	All (n = 89)	ACF (n = 23)	Walk-Ins (n = 66)
Age in years, median (IQR)	34 (23–49) Missing data 64/969	38 (26–52) Missing data 39/460	31 (21–46) Missing data 25/509	33 (23–44) Missing data 2/89	33 (25–49) Missing data 2/23	32.5 (21–42.75) Missing data 0/66
Gender (female), *n* (%)	516/955 (54.0)	249/460 (54.1)	267/495 (53.9)	33/89 (37.1)	6/23 (26.1)	27/66 (40.9)
Sub-county of origin, *n* (%)	K. Central 22/914 (2.4) K. East 76/914 (8.3) K. North 185/914 (20.2) K. West 631/914 (69.0)	K. Central 22/440 (5.0) K. East 74/440 (16.8) K. North 136/440 (30.9) K. West 208/440 (47.3)	K. Central 0/474 (0) K. East 2/474 (0.4) K. North 49/474 (10.3) K. West 423/474 (89.2)	K. Central 2/89 (2.2) K. East 5/89 (5.6) K. North 19/89 (21.3) K. West 63/89(70.8)	K. Central 2/23 (8.7) K. East 3/23 (13.0) K. North 8/23 (34.8) K. West 10/23 (43.5)	K. Central 0/66 (0) K. East 2/66 (3.0) K. North 11/66 (16.7) K. West 53/66 (80.3)

## Data Availability

Data are contained within the article or supplementary material.

## Supplementary Materials

The following supporting information can be downloaded at https://www.mdpi.com/article/10.3390/microorganisms11051280/s1. Table S1: Kajiado County’s demography and TB epidemiological data; Figure S1: Map of the sites involved in the ATI-TB project; Table S2: Raw data for positive and presumptive TB cases.
