# Supplementary material for: Burden of Pulmonary Rifampicin-Resistant Tuberculosis in Kajiado, Kenya: An Observational Study
Source: Microorganisms. 2023 May 13;11(5):1280. doi: 10.3390/microorganisms11051280 (PMC10220680; doi:10.3390/microorganisms11051280)
Supplement: Supplementary file 1 [file microorganisms-11-01280-s001.zip › Figure S1.pdf]

Figure S1. Map of the sites involved in the ATI-TB project

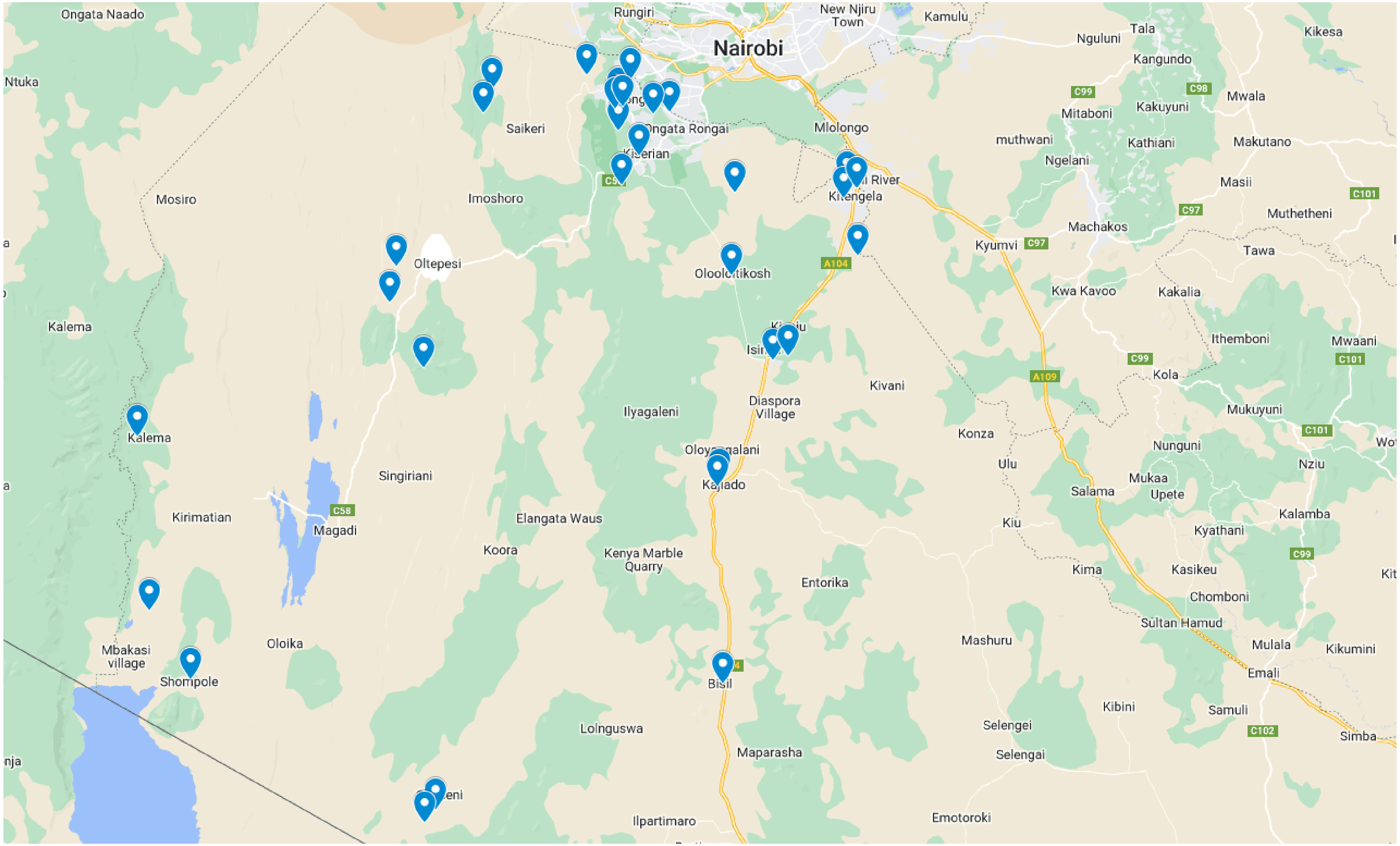

The communities involved in the “raising awareness and active case finding campaigns” are marked in blue. Two new Xpert MTB/RIF machines were also located in Ngong and Oltepesi and were available for routinely testing of *walk-in* patients.
