# Supplementary material for: Burden of Pulmonary Rifampicin-Resistant Tuberculosis in Kajiado, Kenya: An Observational Study
Source: Microorganisms. 2023 May 13;11(5):1280. doi: 10.3390/microorganisms11051280 (PMC10220680; doi:10.3390/microorganisms11051280)
Supplement: Supplementary file 1 [file microorganisms-11-01280-s001.zip › Table S1.pdf]

TABLE S1. Kajiado County's demography and TB epidemiological data

**Kajiado demographic data***	
Population	1,107,296
Population >=15 ys	646,661
Annual population growth (estimates)	5.5%
Subcounties population: Kajiado North	304,404
Kajiado East	271,576
Kajiado Central	159,520
Kajiado West	181,622
Kajiado South	190,174
Age distribution of the population (male:female ratio): <15 ys	41.6% (1.02:1)
15-24 ys	20.9% (0.85:1)
25-34 ys	17.9% (1.04:1)
35-44 ys	9.9% (1.18:1)
45-54 ys	5.0% (1.16:1)
>54 ys	4.7% (1.02:1)
**Kajiado TB data from TIBU, June 2021 - May 2022^**	
Total pulmonary TB incidence, all ages (bacteriologically+clinically diagnosed)	108:100,000 population (1,331:1,232,448)
Pulmonary TB incidence, all ages (bacteriologically diagnosed)	76:100,000 population (936:1,232,448)
Total pulmonary TB incidence, >=15 ys (bacteriologically+clinically diagnosed)	172:100,000 population (1,238:719,750)
Pulmonary TB incidence, >=15 ys (bacteriologically diagnosed)	126:100,000 population (907:719,750)
% Rifampicin resistance among pulmonary bacteriologically confirmed cases	1.0% (9:936)
% Rifampicin resistance among all pulmonary notified cases	0.7% (9:1,331)
% of cases with HIV-coinfection	23.4%
**Kajiado TB data from TIBU, 2019^**	
Total pulmonary TB incidence, all ages (bacteriologically+clinically diagnosed)	128:100,000 population (1,412:1,107,296)
Pulmonary TB incidence, all ages (bacteriologically diagnosed)	88:100,000 population (976:1,107,296)
Total pulmonary TB incidence, >=15 ys (bacteriologically+clinically diagnosed)	201:100,000 population (1,301:646,661)
Pulmonary TB incidence, >=15 ys (bacteriologically diagnosed)	146:100,000 population (945:646,661)
% Rifampicin resistance among pulmonary bacteriologically confirmed cases	1.2% (12:976)
% Rifampicin resistance among all pulmonary notified cases	0.8% (12:1,412)
% of cases with HIV-coinfection	23.8%

* Data shown are primarily derived from 2019 Kenya Population and Housing Census (reference 22 in the main text); annual population growth estimates and age distribution were not available from 2019 Census and are therefore derived from the County Integrated Development Plan 2018-2022 (reference 21 in the main text).
^The epidemiological variables reported here are inferred from data recorded in TIBU (National TB Reporting Platform), a platform developed by the Kenyan Ministry of Health (reference 23 in the main text).
